# Prediction of kidney transplant outcome based on different DGF definitions in Chinese deceased donation

**DOI:** 10.1186/s12882-019-1557-x

**Published:** 2019-11-13

**Authors:** Xiao-jun Hu, Jin Zheng, Yang Li, Xiao-hui Tian, Pu-xun Tian, He-li Xiang, Xiao-ming Pan, Chen-guang Ding, Xiao-ming Ding, Wu-jun Xue

**Affiliations:** 1 Department of Renal Transplantation, Nephropathy Hospital, The First Affiliated Hospital of Xi’an Jiaotong University, Xi’an, 710061 Shaanxi China; 2 Institute of Organ Transplantation, Xi’an Jiaotong University, Xi’an, 710061 Shaanxi China

**Keywords:** Delayed graft function, Donation after cardiac death, Kidney transplant outcome, Definitions

## Abstract

**Background:**

Delayed graft function (DGF) is an important complication of kidney transplantation and can be diagnosed according to different definitions. DGF has been suggested to be associated with the long-term outcome of kidney transplantation surgery. However, the best DGF definition for predicting renal transplant outcomes in Chinese donations after cardiac death (DCDs) remains to be determined.

**Method:**

A total of 372 DCD kidney transplant recipients from June 2013 to July 2017 in the First Affiliated Hospital of Xi’an Jiaotong University were included in this retrospective study to compare 6 different DGF definitions. The relationships of the DGF definitions with transplant outcome were analyzed, including graft loss (GL) and death-censored graft loss (death-censored GL). Renal function indicators, including one-year estimated glomerular filtration rate (eGFR) and three-year eGFR, and were compared between different DGF groups.

**Results:**

The incidence of DGF varied from 4.19 to 35.22% according to the different DGF diagnoses. All DGF definitions were significantly associated with three-year GL as well as death-censored GL. DGF based on requirement of hemodialysis within the first week had the best predictive value for GL (AUC 0.77), and DGF based on sCr variation during the first 3 days post-transplant had the best predictive value for three-year death-censored GL (AUC 0.79). Combination of the 48-h sCr reduction ratio and classical DGF can improve the AUC for GL (AUC 0.85) as well as the predictive accuracy for death-censored GL (83.3%).

**Conclusion:**

DGF was an independent risk factor for poor transplant outcome. The combination of need for hemodialysis within the first week and the 48-h serum creatinine reduction rate has a better predictive value for patient and poor graft outcome.

## Background

Donation after cardiac death (DCD) has become the main source of organ transplantation in China since the use of organs from executed prisoners was forbidden in 2015 [1]. Because of the organ supply shortage, the use of kidneys from expanded criteria donors (ECDs) is growing rapidly [2]. According to previous literature [3], ECDs were defined as donors aged 60 years and older and those aged 50–59 years with at least two of three other conditions (cerebrovascular cause of death, terminal creatinine> 1.5 mg/dl, and hypertension). Compared with the risk from other organ sources, the risk of delayed graft function (DGF) in DCD and ECD transplantation is obviously higher [4, 5].

DGF is used to describe the status of transplanted kidneys that fail to function immediately after transplantation and is an important complication of kidney transplantation. There is no consensus in the literature about how to define DGF. The straightforward United Network for Organ Sharing definition of DGF is the need for at least one dialysis treatment in the first week after transplantation (classical DGF) [6]. As reported in previous literature [7], DGF increases the risk of chronic allograft failure and acute rejection [8, 9], which worsens allograft and patient survival [10–13].

DGF is considered to have a close relationship with ischemia-reperfusion injury (I/RI). Acute kidney injury (AKI) caused by I/RI is thought to be the most common reason an allograft fails to function immediately. The dialysis-based definition of DGF is universally accepted, but it is difficult for physicians to differentiate from other causes of early graft dysfunction which require dialysis, such as hyperacute rejection, calcineuin inhibitor nephrotoxicity, vascular complications and urinary complications. All the complications mentioned above have similar manifestations including hypervolemia, hyperkalemia, elevation of nitrogenous substances [14, 15]. Thresholds for dialysis differ among clinicians, and the definition of DGF according to the dialysis requirement is subjective. Thus, patients may be diagnosed with DGF even though their allograft function is considerable.

The manifestation of DGF not only means a need for dialysis but also reflects the oliguresis, slowly decreased serum creatinine (sCr) and other conditions. There are different definitions of DGF in the literature for diagnosing DGF based on the sCr reduction ratio or urine output after surgery, which are much more objective and measurable. In 1998, Giral-Classe M et al. [16] defined DGF as occurring when the time required for the kidney to reach a creatinine clearance > 10 ml/min is greater than 1 week. In 2000, Boom H et al. [17] proposed the definition as sCr that increases or remains unchanged or decreased < 10%/day during 3 consecutive days after transplantation. In 2005, Thorne-Tjomsland G [18] defined DGF as sCr > 2.5 mg/dl on day 7 or the need for post-transplant hemodialysis. Nickerson defined DGF as failure of creatinine to decline in the first 48 h in the absence of rejection [19]. Shoskes defined DGF as urine output < 75 ml/h in the first 48 h or failure of sCr to decrease by 10% in the first 48 h [20]. All definitions mentioned above were based on three essential elements, including the hemodialysis requirement, sCr and urine output post-transplant. The purpose of this study was to compare the correlations of these objective DGF definitions with transplant outcomes in Chinese DCD kidney transplants and to identify superior methods for diagnosing DGF.

## Methods

### Study cohort and ethics statement

For this observational cohort study, we collected data from deceased donors in a single center from May 2013 to June 2016. The study cohort was approved by the clinical research institution of the First Affiliated Hospital of Xi’an Jiaotong University and was conducted in accordance with the principles of the Declaration of Helsinki. In this study, no organs were obtained from prisoners. Organs were obtained by the Organ Procurement Organization (OPO) of the First Affiliated Hospital of Xi’an Jiaotong University and were allocated by China Organ Transplant Response System. The process of organ procurement and the transplant surgeries were approved by the Ethics Committee of the First Affiliated Hospital of Xi’an Jiaotong University and the Red Cross Society of Shaanxi Province. We excluded recipients < 16 years old and recipients of dual and en-bloc kidneys and multi-organ transplants. Recipients were followed up for a mean period of 1085.97 ± 262.29 days after transplantation.

### Immunosuppression and postoperative management

The basic immunosuppressive regimen used at the First Affiliated Hospital of Xi’an Jiaotong University included cyclosporine or tacrolimus, mycophenolate mofetil (MMF), and prednisone. Rabbit antithymocyte globulin (rATG) (1.25–1.50 mg/ (kg·d), intravenously) was administered for induction therapy on the day of surgery and was then tapered until discontinuation on postoperative day 5. Cyclosporine (6 mg/kg per day) or tacrolimus (0.06 mg/kg per day) was started with MMF (2000 mg/d) to maintain appropriate trough levels in the blood. Methylprednisolone was administered (500 mg i.v.) on the day of surgery, tapered along with the rATG and then replaced by prednisone (10 mg/d). Supplementary tapered methylprednisolone was administered for three to five consecutive days when acute rejection was suspected with or without pathological evidence.

### Exposure variables

We categorized recipients into different groups according to various literature-based DGF definitions, as shown in Table 1. The sCr and urine output were recorded every day. Creatinine clearance on day 7 post-transplant was calculated according to the Modification of Diet in Renal Disease (MDRD) equation [21–23]: [Sex*((140-Age in years)/(SCr in mg/dl))*(kg/72)], where Sex = 1 for male, 0.85 for female, and ignoring sCr after dialysis post-transplant.

**Table 1:** Literature-based DGF definitions and DGF incidence

Abbreviation	Time	Definition	Incidence
Classical DGF	–	the need for at least one dialysis treatment in the first week after Tx	19.89%
Boom DGF	2000	sCr increasing, remaining unchanged or decreasing < 10%/day during 3 consecutive days after Tx	35.22%
Giral DGF	1998	greater than 1-week period required for the kidney to reach creatinine clearance > 10 ml/min	4.19%
Nick DGF	1998	failure of creatinine to decline in the first 48 h in the absence of rejection	18.16%
Shoskes DGF	1995	urine output < 75 ml/h in first 48 h or failure of sCr to decrease by 10% in the first 48 h	15.49%
Turk DGF	2005	sCr > 2.5 mg/dl on day 7 or the need for post-transplant hemodialysis	28.49%

*Tx* transplant surgery

### Outcome variables

We set graft loss (GL) as a primary dichotomous outcome. The current definition for GL used by the U.S. registry and regulatory bodies overseeing transplantation, including UNOS, the Scientific Registry of Transplant Recipients (SRTR) and the Centers for Medicare and Medicaid Services (CMS), encompasses a composite of both GL (resumption of maintenance dialysis, eGFR less than 10 ml/min/1.73 m^2^, graft excision or retransplantation) and death [23]. Graft survival was defined as living recipients with a functional graft. Transplant outcome included GL as well as death-censored GL. We calculated the estimated glomerular filtration rate (eGFR) from clinical sCr measurements at specified time points via the MDRD Study Equation [24].

### Statistical analysis

Continuous variables are reported as the means±SD (standard deviation), and categorical variables are reported as frequencies (percentages). GL was assessed as the primary outcome. Secondary outcomes, including 12-month and 3-year eGFR, were compared between the DGF and non-DGF groups according to various literature-based DGF definitions using the Mann-Whitney U test.

For survival analysis, GL was estimated via Kaplan-Meier survival curves. The impacts of various literature-based DGF on GL were analyzed using the log-rank test. Multivariate Cox regression models were performed to estimate the relationship between each DGF diagnosis approach and GL after adjustment for different relevant variables according to previous literature, including donor age (years), donor hypertension history, cold ischemia time, and donor terminal sCr.

A receiver operating characteristic curve (ROC) was calculated to compare the predictive value of the clinical status based on different DGF definitions. Sensitivity, specificity, and diagnostic accuracy were calculated to further compare definitions. A two-sided *P*-value of 0.05 was considered statistically significant.

Statistical analysis was performed using R software.

## Results

### Cohort description

All recipients in our cohort received DCD organs. The study cohort consisted of 372 recipients. The median follow-up time was 1209 days after transplantation. All recipients were divided into a non-graft loss group (NGL group) and a GL group.

All recipients included those who accepted kidney transplant surgery for the first time. The baseline information on the donors and recipients is summarized in Table 2. The donors and their characteristics were not significantly different between the two groups. The mean recipient ages in the NGL and GL groups were 36.39 ± 9.39 years and 37.59 ± 10.79 years, respectively. Most of the recipients were male and chose hemodialysis before transplant surgery. The mean dialysis durations were 22.61 ± 21.83 months in the NGL group and 21.98 ± 19.44 months in the GL group, with no difference between the groups via the Mann-Whitney U test.

**Table 2:** Recipient and donor characteristics (study cohort)

	NGL Group	GL Group	*P*-value
Parameter	*n* = 314	*n* = 58	
Recipient characteristics			
Age (mean ± SD, years)	36.39 ± 9.39	37.59 ± 10.79	0.383
Male/female ratio	220/94	50/8	0.118
BMI (mean ± SD, kg/m^2^)	20.83 ± 3.15	20.46 ± 3.69	0.427
ABO blood type (n,%)			
A	95 (30.3%)	20 (35.1%)	0.414
B	95 (30.3%)	21 (36.8%)	
AB	25 (8.0%)	4 (7.0%)	
O	99 (31.5%)	12 (21.1%)	
HLA mismatch (mean ± SD)	1.82 ± 1.00	1.84 ± 1.06	0.984
Dialysis			
PD/HD ratio	29/275	6/49	0.809
time before Tx (mean ± SD, months)	22.61 ± 21.83	21.98 ± 19.44	0.838
PRA positive (n,%)	24 (7.6%)	8 (13.8%)	0.201
Donor characteristics			
Age (mean ± SD, years)	40.18 ± 16.22	38.37 ± 19.09	0.804
Male/female ratio	145/36	24/7	0.918
BMI (mean ± SD, kg/m^2^)	21.76 ± 3.70	21.15 ± 3.44	0.594
Cause of death (n,%)			
Trauma	91 (50.0%)	19 (61.3%)	0.594
Cerebrovascular disorders	64 (35.2%)	9 (29.0%)	
Hypoxic ischemic encephalopathy	13 (7.1%)	2 (6.5%)	
Tumor	10 (5.5%)	0 (0.0%)	
Others	4 (2.2%)	1 (3.2%)	
History of hypertension (n,%)	42 (23.1%)	8 (25.0%)	0.992
Terminal sCr (mean ± SD, μmol/L)	107.99 ± 76.80	99.53 ± 66.50	0.565
Cold ischemia time (mean ± SD, hours)	6.48 ± 3.00	6.01 ± 2.95	0.417
Warm ischemia time (mean ± SD, mins)	7.84 ± 3.88	8.74 ± 3.29	0.221
ECD(n,%)	29 (15.9%)	8 (25.0%)	0.319

*HLA* human leucocyte antigen, *Tx* transplant surgery, *sCr* serum creatinine, *PD* peritoneal dialysis, *HD* hemodialysis, *PRA* panel reactive antibody, *ECD* expanded criteria donors; ^a^At the time of transplantation; Continuous variables were compared via the Mann-Whitney U test, and categorical variables were compared via the Chi-square test

The mean donor ages were 40.18 ± 16.22 years in the NGL group and 38.37 ± 19.09 years in the GL group. A total of 37 donors were defined as ECD: 29 in the NGL group (15.9%) and 8 in the GL group (25.0%). Brain trauma was the most common cause of death for donors in the NGL group (50.0%) and the GL group (61.3%). A history of hypertension was reported in 23.1% donors in the NGL group and 25.0% donors in the GL group. The mean cold ischemia times were 6.48 ± 3.00 h in the NGL group and 6.01 ± 2.95 h in the GL group (range from 2 to 16 h). The mean warm ischemia times were 7.84 ± 3.88 min in the NGL group and 8.74 ± 3.29 min in the GL group (range from 3 to 24 min). The mean terminal sCr levels of donors before procurement were 107.99 ± 76.80 μmol/L in the NGL group and 99.53 ± 66.50 μmol/L in the GL group.

### Incidence of DGF

Table 1 shows different DGF incidences in our cohort. Boom DGF, defined based on sCr change during first 3 days post-transplant, had the highest incidence of 35.22%. Giral DGF, defined based on the renal function recovery time, had the lowest incidence of 4.19%. Classical DGF, Nick DGF, Turk DGF and Shoskes DGF had DGF incidences of 19.89, 18.16, 28.49 and 15.49%, respectively.

### Effect of DGF on 3-year graft outcome

The distribution of reasons for GL is presented in Table 3; 24 GL patients died with a functional allograft, and 18 of those patients lost the graft for chronic reasons.

**Table 3:** Distribution of recipient GL in our cohort

Graft loss reason	N (%)
Patient death	
Severe pulmonary infection	9 (15.5%)
Gastrointestinal bleeding	2 (3.4%)
Multiple organ dysfunction syndrome	5 (8.6%)
Cerebrovascular event	1 (1.7%)
Unknown cause	7 (12.1%)
Graft excision	
Rupture of graft artery	10 (17.2%)
Thrombosis	4 (6.9%)
Urinary tract obstruction	2 (3.4%)
Chronic graft failure	18 (31.0%)

**Fig. 1:**
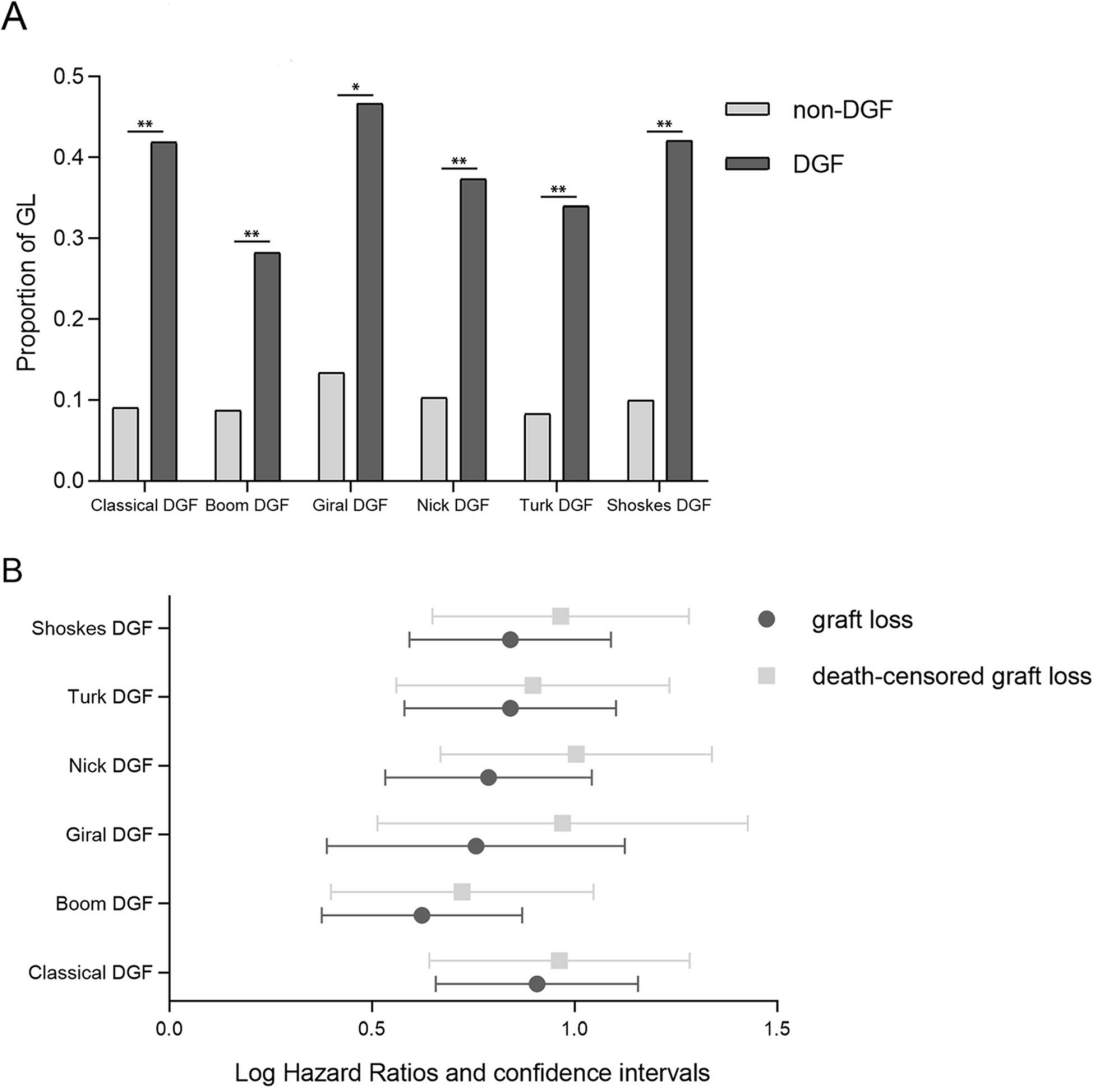
**a**. Three-year post-transplant GL proportions in the DGF and non-DGF cohorts. **b**. A multivariate Cox proportional hazards model adjusting for donor age, cold ischemia time, HLA mismatch, donor hypertension history and donor terminal sCr. The hazard ratio for GL and death-censored GL in patients with DGF were compared with each other. Dots represent the logarithms of the hazard ratios. Segments represent the 95% confidence intervals

We examined the association between DGF and GL and death-censored GL via multivariate Cox regression models to determine whether any specific DGF definition was able to predict graft failure more effectively. The three-year graft survival of our cohort was 84.45%. As shown in Fig. 1a, graft survival for kidneys that fulfilled the definition of DGF was associated with more than 3 times the risk of GL, with a significant difference. All DGF definitions were apparently associated with three-year GL and three-year death-censored GL of DCD kidneys via the multivariate Cox proportional risk analysis (Fig. 1b). Classical DGF was the best definition for three-year GL prediction for its largest HR value, 8.09. Nick DGF was the best definition for three-year death-censored GL for its largest HR value, 10.10.

**Fig. 2:**
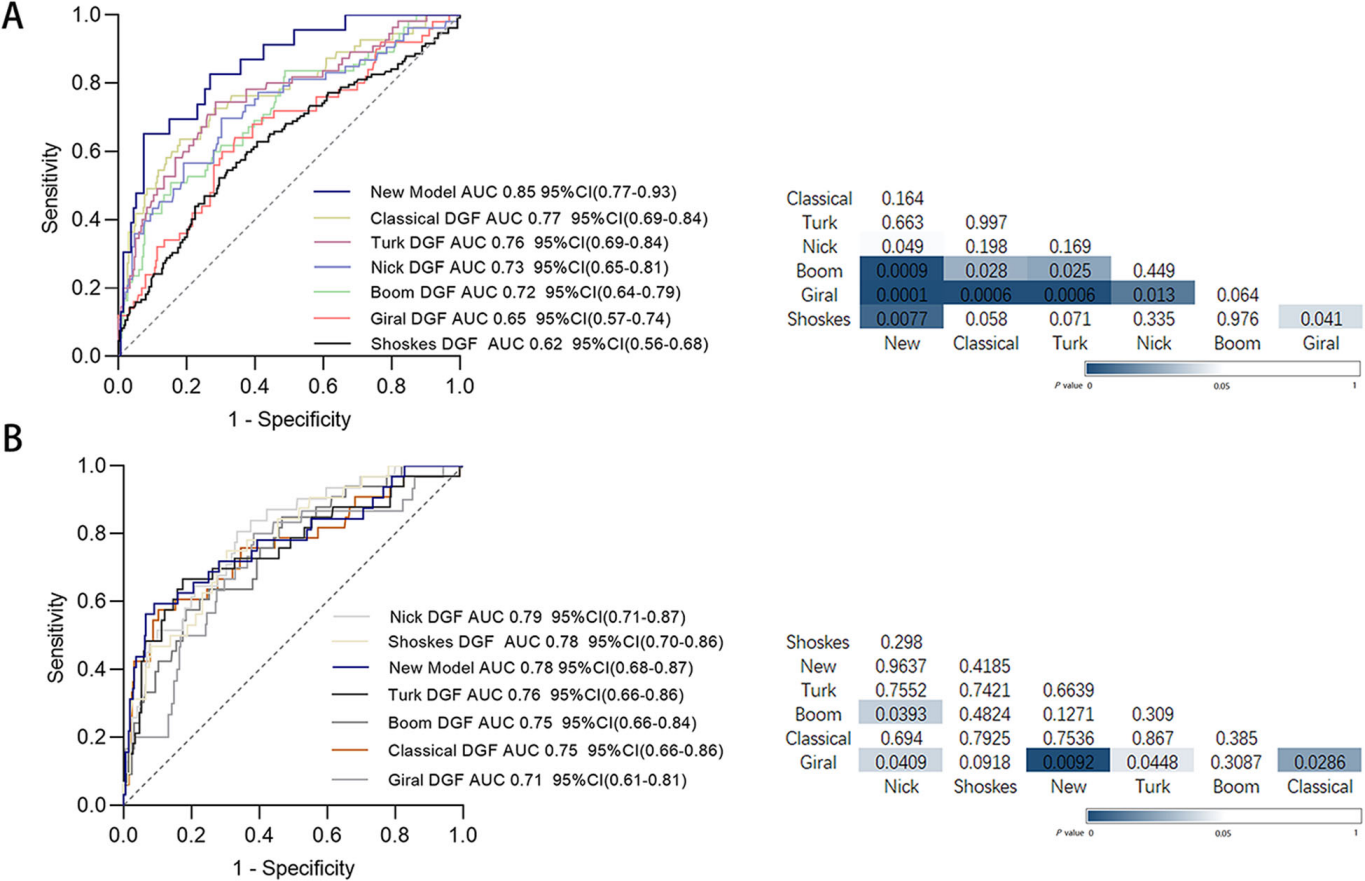
Evaluation of the predictive power of the seven DGF definitions for three-year GL(**a**) and death-censored GL(**b**) by a receiver operating characteristic (ROC) curve analysis using multivariate logistic regression models adjusting for donor age, cold ischemia time, HLA mismatch, donor hypertension history and donor terminal sCr. All DGF definition ROC curves were compared with each other via DeLong’s test. The AUCs of different DGF models declined from the top to the bottom as well as from the left to the right. Values and colored boxes in the grid represent the *P*-values of DeLong’s test. Blue shading represents the significance of the difference between ROC curves

ROC curves were applied to examine the power of DGF for predicting GL in our cohort (Fig. 2a). The AUC values ranged from 0.62 to 0.77. Classical DGF was the best fit for predicting GL with the largest AUC value of 0.77, significantly better than Giral and Boom DGF (0.77 vs. 0.65, 0.77 vs. 0.72, Fig. 2a). For predicting three-year death-censored GL (Fig. 2b), AUCs ranged from 0.71 to 0.79, and Nick DGF had the best performance, with an AUC value of 0.79, significantly better than both Boom and Giral DGF (0.79 vs. 0.71, 0.79 vs. 0.75). Giral DGF had the lowest accuracy for GL and death-censored GL. Thus, Giral and Boom DGF were inferior to other DGF definitions for predicting three-year graft outcome.

### Effect of DGF on post-transplant renal function

One-year eGFR was available for 259 of 372 (69.6%) of patients. The one-year graft functions of kidneys that fulfilled the criteria for DGF were significantly poorer in all DGF definitions except Giral DGF (Table 4), albeit there was still a nonsignificant trend toward lower 1-year eGFR (73 vs. 65 ml/min/1.73 m^2^; *P* = 0.815). Three-year eGFR was available for 290 of 372 (80.0%) of patients. The association between DGF definition with three-year eGFR was similar to the one-year graft function (Table 4). The difference in three-year eGFRs between Giral DGF-positive patients and Giral DGF-negative patients was not significant (74 vs. 65 ml/min/1.73 m^2^; *P* = 0.305). Thus, DGF was associated with poorer renal function post-transplant, no matter how it was defined.

**Table 4:** Effects of DGF on 1- and 3-year graft function

Definition of DGF	1-year eGFR			3-year eGFR		
	DGF-	DGF+	*P*-value	DGF-	DGF+	*P*-value
Classical DGF	76 (61–89)	64 (52–75)	**< 0.001**	75 (61–92)	65 (51–82)	**0.005**
Boom DGF	78 (64–90)	65 (55–77)	**< 0.001**	76 (61–93)	67 (55–84)	**0.007**
Giral DGF	73 (60–88)	65 (58–75)	0.815	74 (59–90)	65 (53–75)	0.305
Nick DGF	76 (64–89)	61 (50–72)	**< 0.001**	75 (61–92)	63 (51–79)	**0.001**
Turk DGF	78 (65–90)	62 (51–73)	**< 0.001**	76 (62–93)	62 (50–79)	**< 0.001**
Shoskes DGF	75 (62–88)	61 (50–76)	**0.001**	75 (61–91)	63 (54–80)	**0.024**

eGFR (median [interquartile range] by definition of DGF; eGFR in ml/min/1.73 m^2^ and compared via the Mann-Whitney U test)

Bold data means significance

### Classical DGF combined with 48 h sCr reduction ratio

The association of the 48 h creatinine reduction ratio when combining classical DGF with GL was tested via the Cox proportional hazards model. It was proved that adding the 48-h sCr reduction ratio to the classical DGF model could improve the AUC for predicting GL as well as death-censored GL (Fig. 2). The AUC value for GL was 0.85, better than all DGF phenotypes (Fig. 2a), with a sensitivity of 71.7%, a specificity of 78.6%, and an accuracy of 73.4% (Table 5). Combining the 48-h sCr reduction ratio with classical DGF was not superior than Nick and Shoskes DGF for predicting three-year death-censored GL when taking the AUC into account (Fig. 2b), but its accuracy was the best (83.3%, Table 5).

**Table 5:** Sensitivity, specificity and diagnostic accuracy of each definition for graft loss for recipients of DCD kidney transplants

	Sensitivity (%)	Specificity (%)	Accuracy (% [95% CI])
GL			
Shoskes DGF	47.2	89.6	78.2 (73.8,82.1)
Boom DGF	50.9	84.6	75.3 (70.6,79.4)
Classical DGF	63.6	81.9	75.3 (70.6,79.4)
New model	71.7	78.6	73.4 (68.7,77.6)
Turk DGF	74.5	71.6	68.3 (63.4,72.8)
Nick DGF	69.8	69.8	65.6 (60.6,70.2)
Giral DGF	64.0	65.9	59.7 (64.5,54.6)
Death-censored GL			
New model	59.4	90.1	83.3 (79.2,86.8)
Classical DGF	57.6	89.72	82.5 (78.3,86.0)
Turk DGF	66.7	82.5	77.2 (72.6,81.1)
Boom DGF	57.6	81.6	75.3 (70.6,79.4)
Shoskes DGF	75.0	69.6	65.9 (60.9,70.5)
Nick DGF	80.6	66.6	64.0 (59.0,68.7)
Giral DGF	80.0	61.6	57.5 (52.5,62.4)

New model, Classical DGF combined with 48-h creatinine reduction ratio

## Discussion

DGF is an important and intricate complication after kidney transplant surgery. Its mechanism is not completely understood. Previous studies have shown that DGF influences the transplant surgery outcome from DCD [25–27]. DGF is universally defined as the need for hemodialysis at 7 days post-transplant. This dialysis-based definition is subjective and has many other causes unrelated to renal function, such as hyperkalemia, volume overload, heart failure and so on [15]. This fact may be a possible reason why different studies about the association of DGF and graft survival have yielded opposite results. Thus, some patients with considerable graft function can be misdiagnosed with DGF. Physicians have put forward some other DGF definitions to fill that gap. This study analyzed 6 DGF definitions based on urine output, creatinine, and necessity of dialysis in the early post-transplant stage and compared their predictive power for the transplant outcome. Furthermore, the present investigation is the first to compare DGF definitions with respect to Chinese DCD transplantation.

Using retrospective cohort data for deceased-donor kidney transplant recipients, we have shown that all DGF definitions were significantly associated with three-year GL and had considerable predictive power for this outcome in the Chinese DCD cohort despite the incidence of DGF fluctuating greatly according to the definition used. DGF was associated with a more than 3-fold three-year GL. This phenomenon could be ascribed to the reduced confidence in the recovery of recipients if they suffered from DGF. In China, kidney transplant surgeries are expensive for most families, and patients usually have great expectations of transplant outcomes. DGF does not occur in China as often as it does in Western countries (20% vs. 70%) [28]. Patients suffer from tremendous psychological pressure if DGF occurs, and transplant outcomes are negatively influenced. Our results were consistent with those of some previous studies [29] and contrasted with the results of others [30]. Decruyenaere et al. [29] found that dialysis-based DGF was significantly associated with graft failure, with hazard ratios (HRs) ranging from 2.87 to 13.73. However, Mallon et al. found that DGF in DCD kidneys was not associated with inferior graft survival but that DGF was an independent risk factor in DBD cohorts. The authors ascribed this difference to the much more severe warm ischemic damage in DCD organs in the UK transplant population. It is possible that warm ischemia promotes the development of acute tubular necrosis, which was thought to be a characteristic of the delayed graft function [31, 32]. The longer the warm ischemia time (WIT) is, the more severe the damage will be. In our population, there were significant differences in WIT. This finding may explain why DGF has a considerable predictive power for transplant outcome in DCD transplantation, in contrast to the results in other countries.

In this study, GL was defined as resumption of maintenance dialysis, eGFR less than 10 ml/min/1.73 m^2^, graft excision or retransplantation and patient death. This definition embraced bad endings for both patient and graft. The ROC curve of classical DGF predicting GL overlapped with other curves except for those of Giral and Boom DGF, both of which were based on sCr variation in the early post-transplant stage. This result illustrated that definitions based on objective indicators such as creatinine were not better than the classical definition. Compared with GL, death-censored GL was a more prominent indicator and could reflect graft outcome post-transplant. Nick DGF based on the 48-h creatinine variation had the best performance in prediction because of its largest AUC value, but its ROC curve overlapped with others. Thus, we combined these top two DGF definitions and proved that this new predictive model had the largest AUC for predicting GL and the best predictive accuracy for death-censored GL. The combination of creatinine- and hemodialysis-based DGF definitions can avoid the problems mentioned above and has superior operability.

Definitions based on urine output in our study were combined with creatinine values (Shoskes DGF). In this study, it was a poor indicator for transplant outcome. Using urine output-based definitions may perplex clinicians because it is not possible to differentiate urine outputs from the native kidney and the graft. Consequently, patients with considerable residual renal function may be misdiagnosed as not having DGF because their original kidneys react well to diuretic treatment in the early post-transplant period. Simultaneously, kidneys with severe acute tubular injury might manifest as nonoliguric renal failure, indicating poor renal function companied with polyuria. In sum, urine-based DGF definitions may exhibit deviations.

The associations of creatinine-based definitions of DGF or early-stage creatinine levels and changes in these levels after transplantation have been discussed many times in the previous literature [33–36]. Using creatinine-based definitions leads to bias as well. Physicians may optimize the status of recipients in whom sCr will be reduced after hemodialysis, and existing DGF may be ignored [37]. In addition, the predictive power of the three-year outcome is controversial. Boom DGF showed relatively poor predictive power, with AUCs of 0.72 for GL and 0.75 for death-censored GL, whereas Giral DGF showed a significantly poorer predictive performance with respect to the three-year transplant outcome, while Nick DGF showed a good performance in predicting death-censored GL. Schnuelle et al. [38] compared the creatinine-based DGF definition with the hemodialysis-based definition and found that the creatinine-based definition had a significant association with graft failure, not in accordance with our results.

Previous studies have shown that post-transplant renal function in the first year predicts long-term kidney transplant survival. The one-year post-transplant eGFR, as the best measurement of renal function, was compared between the DGF and non-DGF groups via a Mann-Whitney U test. In 5 of the 6 definitions, a significant decrease in one-year eGFR was observed if DGF occurred, along with a decrease in the three-year eGFR. The only definition not associated with one-year eGFR was Giral DGF, which is based on renal function recovery time, and it was also the only definition not associated with three-year eGFR post-transplant.

The poor predictive value of Giral DGF, based on whether the period required for the kidney to reach creatinine clearance > 10 ml/min was greater than 1 week, illustrated that renal function recovery time was not a good indicator of defined DGF. This definition had obvious limitations. Many other types of abnormal status may exist or coexist with DGF in the same manner as Giral DGF, such as antibody-mediated rejection, drug-toxic graft dysfunction or primary nonfunction. Unlike Nick DGF, which is based on sCr changes during the first 3 days, Giral DGF is focused much more on the steady result.

In summary, DGF based on the requirement for hemodialysis within the first week had the best predictive value for three-year GL, and DGF based on sCr variation during the first 3 days post-transplant had the best predictive value for three-year death-censored GL. A combination of the 48-h sCr reduction ratio and classical DGF can improve the AUC for GL and the predictive accuracy for death-censored GL.

## Conclusion

DGF was an independent risk factor for bad transplant outcome. A combination of the need for hemodialysis within the first week and the 48-h serum creatinine reduction rate has a better predictive value for patient and graft poor outcomes.

## Data Availability

The data and material used and/or analyzed during the current study are available from the corresponding author.

## Abbreviations

AKI: Acute kidney injury
AUC: Area under curve
CI: Confidence interval
CIT: Cold ischemia time
DBD: Donation after brain death
DCD: Donation after cardiac death
DGF: Delayed graft function
eGFR: Estimated glomerular filtration rate
GL: Graft loss
HD: Hemodialysis
HLA: Human leukocyte antigen
I/RI: Ischemia-reperfusion injury
MMF: Mycophenolate mofetil
NGL: No graft loss.
PD: Peritoneal dialysis
PRA: Panel reactive antibody
rATG: Rabbit antithymocyte globulin
RFRT: Renal function recovery time
ROC: Receiver operating characteristic curve
sCr: Serum creatinine
WIT: Warm ischemia time
